# Beyond the Diaphragm: Grynfeltt-Lesshaft Hernia Presenting With Small Bowel Obstruction

**DOI:** 10.7759/cureus.115296

**Published:** 2026-08-27

**Authors:** Ruth T Samson, Amanda Sekusky

**Affiliations:** 1 Breast Surgery, Montefiore Medical Center, Bronx, USA; 2 Trauma and Acute Care Surgery, Geisinger Wyoming Valley Medical Center, Wilkes Barre, USA

**Keywords:** grynfeltt-lesshaft hernia, incarcerated bowel, posterior abdominal wall hernia, small bowel obstruction, superior lumbar hernia

## Abstract

Lumbar hernias are uncommon defects of the posterolateral abdominal wall and may be difficult to distinguish from other thoracoabdominal abnormalities because of their location and variable appearance on imaging. Superior lumbar, or Grynfeltt-Lesshaft, hernias are particularly rare causes of acute bowel obstruction.

We report the case of a 51-year-old man with obesity and chronic cough who presented with abdominal pain, vomiting, obstipation, dyspnea, and right flank ecchymosis. Computed tomography (CT) obtained before hospital transfer demonstrated herniated small bowel in the right posterolateral thoracoabdominal region, which was concerning for a closed-loop obstruction from a diaphragmatic hernia. Emergent exploratory laparotomy instead demonstrated an intact diaphragm, with a 5-cm defect in the right superior posterolateral abdominal wall immediately inferior to the twelfth rib containing the incarcerated small bowel. The findings were consistent with a superior lumbar hernia. The bowel was viable following reduction, and the defect was closed primarily. This case highlights the diagnostic challenge of superior lumbar hernias and the importance of durable reconstruction when feasible, particularly in patients with factors that may increase intra-abdominal pressure or the risk of recurrence.

## Introduction

Lumbar hernias are rare defects of the posterolateral abdominal wall occurring through areas of relative anatomic weakness between the twelfth rib and iliac crest. They have traditionally been divided into superior lumbar hernias, which occur through the Grynfeltt-Lesshaft triangle, and inferior lumbar hernias, which occur through the Petit triangle [1-4]. Primary lumbar hernias remain uncommon, and the published literature consists predominantly of case reports and small observational series [2,3].

The superior lumbar triangle is generally bordered superiorly by the twelfth rib, medially by the quadratus lumborum or paraspinal musculature, and laterally by the internal oblique muscle. The transversalis fascia and aponeurosis of the transversus abdominis contribute to the floor. The inferior lumbar, or Petit, triangle is bordered inferiorly by the iliac crest, medially by the latissimus dorsi, and laterally by the external oblique muscle, with the internal oblique forming its floor [1,4]. Superior lumbar hernias are reported more frequently than inferior lumbar hernias [2-4].

Lumbar hernias may be congenital or acquired. Acquired defects may occur spontaneously or secondary to trauma, surgery, infection, or disruption of the posterolateral abdominal wall [1-4]. Factors associated with increased intra-abdominal pressure, including obesity and chronic cough, may contribute to the development or progression of primary defects [1-4].

Clinical manifestations range from an asymptomatic flank bulge to pain, incarceration, bowel obstruction, and strangulation. Because of their rarity and deep posterolateral location, lumbar hernias may be mistaken for lipomas, soft-tissue tumors, hematomas, renal pathology, or other abdominal-wall abnormalities [1,5]. Confusion with diaphragmatic pathology may be particularly problematic when the defect lies immediately inferior to the twelfth rib and herniated viscera project toward the lower thorax.

We present a case of an incarcerated right superior lumbar hernia causing small bowel obstruction that was initially interpreted radiographically as a right diaphragmatic hernia. We additionally review the literature concerning the presentation, imaging, complications, and surgical management of lumbar hernias, with emphasis on obstructed and incarcerated cases.

## Case presentation

A 51-year-old man with obesity (body mass index (BMI), 30.3 kg/m²), gastroesophageal reflux disease, anxiety, depression, and motor tics associated with frequent coughing presented with several days of worsening chest discomfort, cough, shortness of breath, abdominal pain, nausea, vomiting, and obstipation. He denied recent trauma, previous abdominal surgery, or a known history of abdominal-wall hernia.

On presentation, he appeared anxious but was not in acute respiratory distress. His blood pressure was 157/85 mmHg, heart rate was 79 beats/minute, respiratory rate was 15 breaths/minute, and temperature was 36.4 °C. His oxygen saturation was 94% while receiving 2 L/minute of supplemental oxygen by nasal cannula.

**Figure 1:**
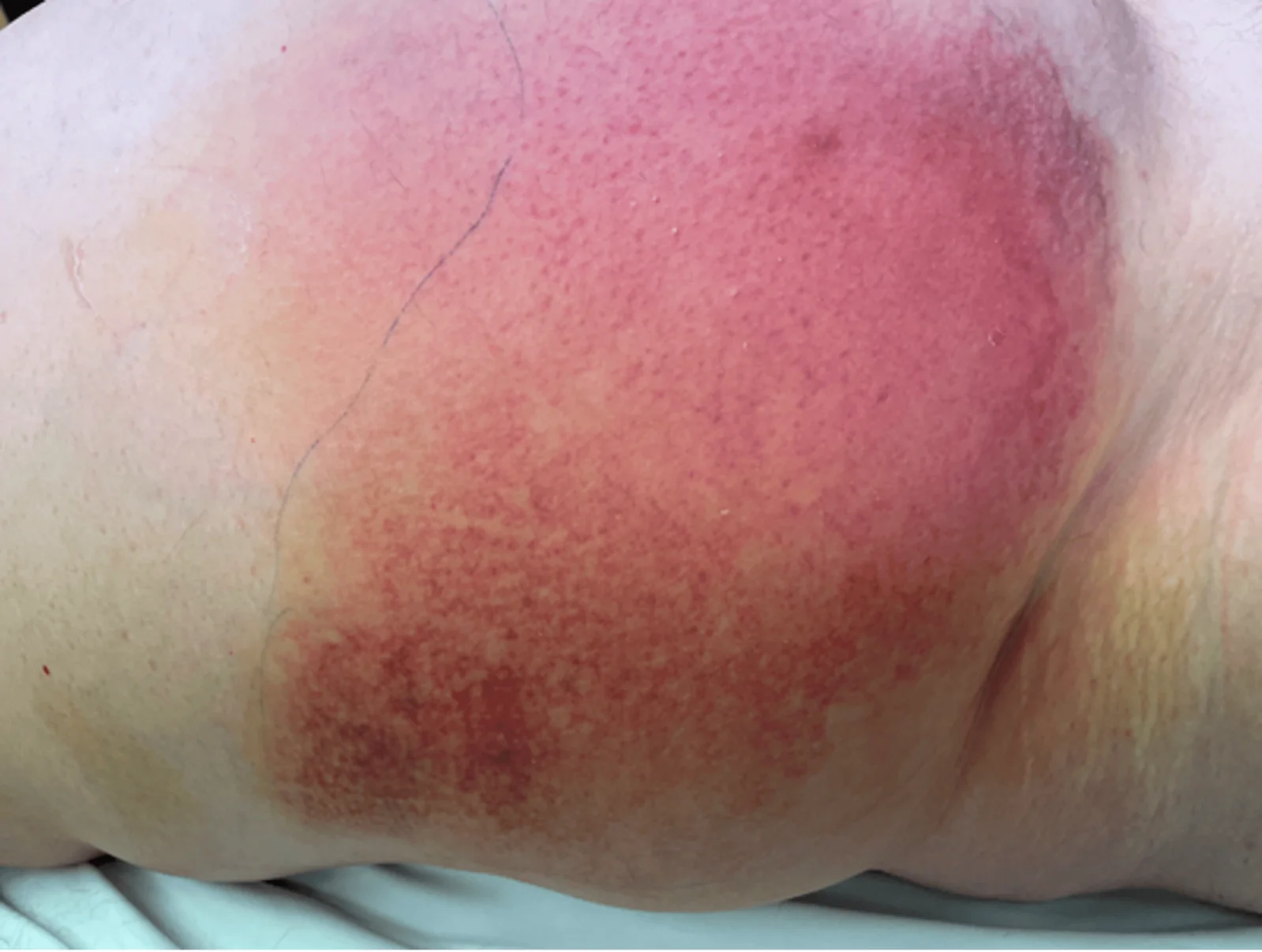
Right flank and right lower abdominal wall ecchymosis noted on presentation

Abdominal examination demonstrated moderate distention and tenderness. Right flank ecchymosis extended toward the mid-abdomen and right lower quadrant (Figure 1). Laboratory evaluation demonstrated leukocytosis with a white blood cell count of 22,000/µL. Serum lactate was within the normal range.

**Figure 2:**
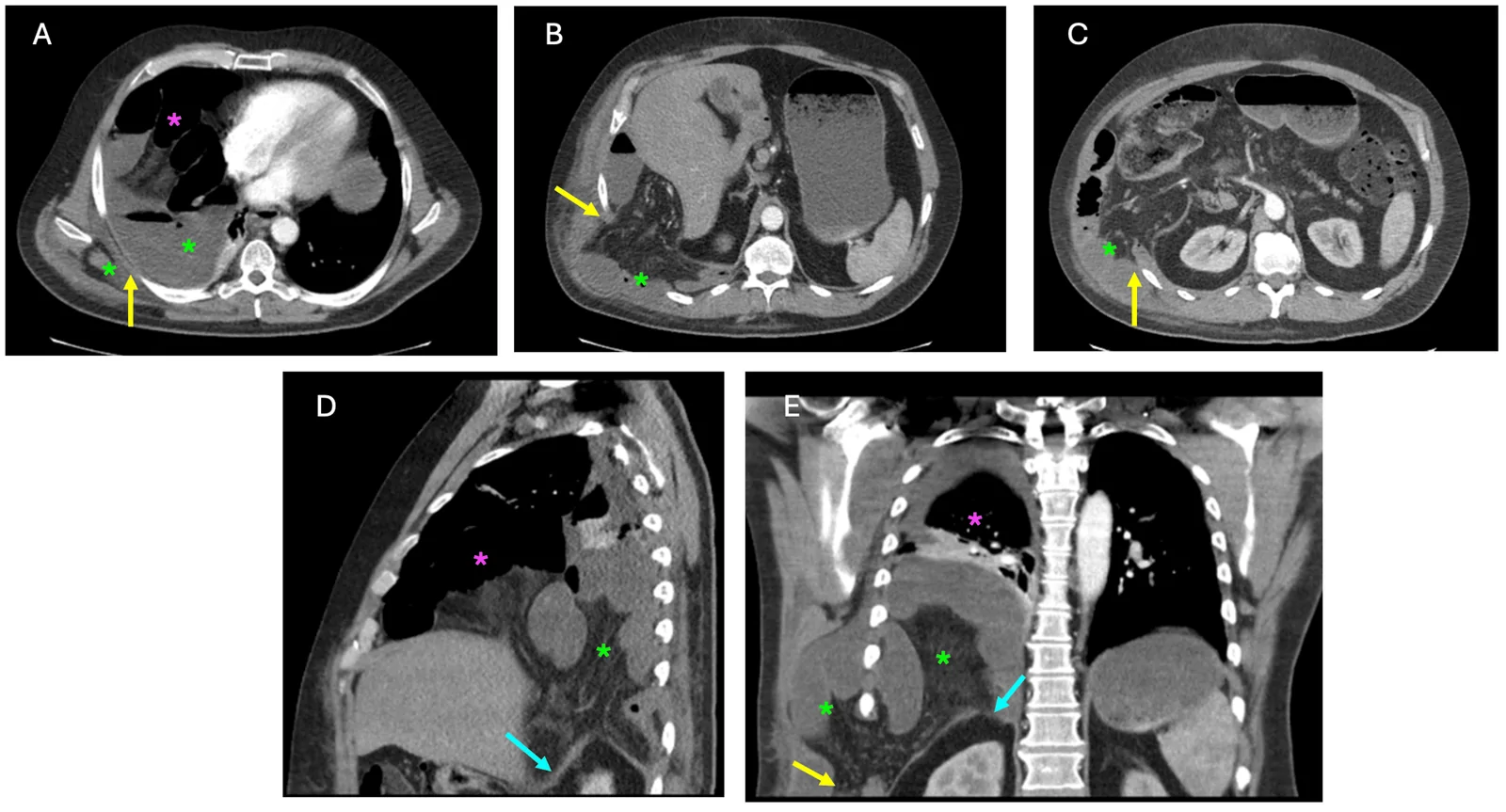
CT findings of the right superior lumbar hernia Axial (A–C), sagittal (D), and coronal (E) contrast-enhanced CT images demonstrate the right posterolateral abdominal wall hernia. Yellow arrows indicate the hernia defect; green asterisks indicate herniated bowel/hernia contents with surrounding fluid; cyan arrows indicate the intact diaphragm; and magenta asterisks indicate the adjacent lung. Multiplanar images (D–E) demonstrate the relationship of the hernia to the intact diaphragm.

Computed tomography of the chest and abdomen obtained at an outside facility demonstrated multiple loops of dilated small bowel extending into the right posterolateral thoracoabdominal region with surrounding fluid. The bowel distal and proximal to the hernia defect appeared decompressed. The findings were interpreted before transfer as a large right diaphragmatic hernia containing small bowel (Figure 2).

The imaging findings were concerning for a closed-loop small bowel obstruction through the suspected diaphragmatic defect. The right flank ecchymosis was also considered a possible external manifestation of the underlying retroperitoneal process, raising concern for ischemia or hemorrhage related to the incarcerated hernia, although its precise etiology could not be established. The patient was therefore consented and taken emergently for exploratory laparotomy with possible thoracic exploration and chest tube placement.

A nasogastric tube was immediately placed for gastric decompression. Following induction of general anesthesia, the patient was prepared and draped from the right hemithorax to the pubic symphysis to permit either an abdominal or thoracic approach if required. A midline laparotomy was performed. A small volume of ascitic fluid was encountered. Exploration of the right upper abdomen demonstrated incarcerated small bowel extending superiorly and posteriorly through a large defect beneath the right rib cage. The incarcerated bowel was carefully reduced with blunt dissection into the abdominal cavity. The reduced small bowel was dilated and congested but remained viable, with no evidence of ischemia or necrosis; therefore, resection was not required.

**Figure 3:**
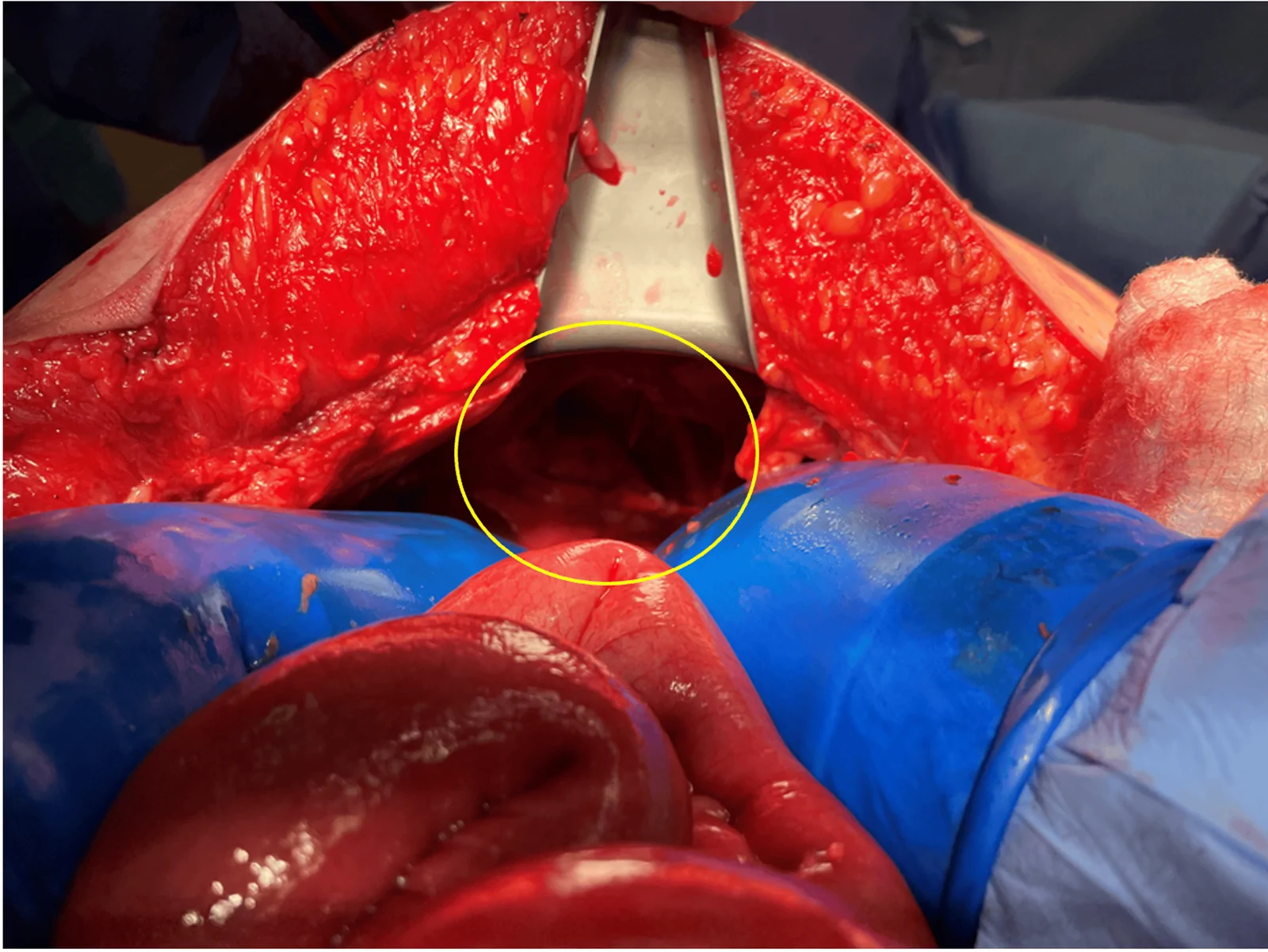
Intraoperative appearance of the right posterolateral abdominal wall defect Following reduction of the incarcerated small bowel, an approximately 5-cm defect was identified in the right superior posterolateral abdominal wall beneath the rib cage (yellow circle). The photograph demonstrates the operative location of the defect; however, the individual muscular boundaries of the superior lumbar triangle are not clearly delineated in this image.

The defect was examined and localized to the right superior posterolateral abdominal wall, immediately inferior to the twelfth rib, measuring approximately 5 cm (Figure 3). The diaphragm appeared intact. Although the individual muscular boundaries of the superior lumbar triangle were not documented in sufficient detail to permit retrospective delineation, the location of the defect and integrity of the diaphragm were consistent with a superior lumbar rather than a diaphragmatic hernia.

Several factors influenced the hernia repair technique, including bowel viability, defect location, operative exposure, and the need for a durable repair. Although mesh reinforcement was considered, the bowel remained markedly dilated, limiting operative exposure. The superior posterolateral location of the defect and the patient's body habitus further complicated mesh placement and secure fixation. Given these technical limitations, definitive mesh reinforcement was not considered feasible during the index operation. The defect was therefore closed primarily with a running #1 polypropylene suture, incorporating the intact and viable muscle on either side of the defect.

The abdomen was then irrigated and closed in the standard fashion. Although the diaphragm appeared intact, the superior extent of the hernia made pleural communication or inadvertent pleural violation difficult to exclude. A 24-French right thoracostomy tube was therefore placed through the fifth intercostal space and connected to −20 cm H₂O suction. The chest tube demonstrated no air leak, and drainage appeared serous.

Postoperatively, the patient’s respiratory status and gastrointestinal recovery were monitored. Nasogastric decompression was maintained until bowel function returned, after which his diet was progressively advanced. The chest tube remained without evidence of an air leak. Chest radiography showed an expanded lung with minimal pleural effusion. On postoperative day five, the chest tube was inadvertently removed. Subsequent chest radiography demonstrated residual pleural effusion without a clinically significant pneumothorax. As the patient's respiratory status remained stable, the chest tube was not replaced. The patient recovered and was discharged home.

At the two-week follow-up, the patient was doing well without clinical signs of hernia recurrence. Approximately four months after the index operation, the patient was evaluated at another institution for intermittent right-sided abdominal discomfort and back pain. Available records documented recurrence of the right lumbar hernia with a plan for definitive hybrid open and robotic retromuscular mesh reconstruction. The outside imaging was not available for review, and the records did not provide sufficient detail on the size or contents of the recurrent hernia or whether the planned reconstruction was performed.

## Discussion

Lumbar hernias are uncommon defects of the posterolateral abdominal wall, and the available literature consists largely of case reports and small retrospective series [2,3]. In a systematic review of primary lumbar hernias, van Steensel et al. identified 14 studies comprising 312 patients and emphasized the limited quality of evidence available to guide diagnosis and treatment [2].

Lumbar hernias may be congenital or acquired, with acquired defects occurring primarily after trauma, surgery, infection, or other disruption of the posterolateral abdominal wall [1-4]. Obesity and conditions associated with chronically increased intra-abdominal pressure are recognized contributing factors [1-4]. In our patient, obesity and repetitive coughing may have increased mechanical stress on the posterolateral abdominal wall, although causality cannot be established.

Although lumbar hernias commonly present as a flank mass or discomfort, bowel obstruction, incarceration, and strangulation are recognized complications [5-9]. Scheffler et al. reported an incarcerated Grynfeltt-Lesshaft hernia causing acute bowel obstruction, with CT demonstrating small bowel within the superior lumbar defect [6]. Ka et al. and Fokou et al. similarly described strangulated or incarcerated lumbar hernias presenting with intestinal obstruction [7,8]. Stupalkowska et al. reported a Grynfeltt-Lesshaft hernia initially considered a lipoma that subsequently presented with acute bowel obstruction [5]. More recently, Patil et al. described strangulated inferior lumbar herniation resulting in gangrenous ileum requiring bowel resection [9].

A notable feature of this case was the initial interpretation of the defect as a diaphragmatic hernia. CT is the principal imaging modality for lumbar hernias and can define the abdominal wall defect, hernia contents, associated complications, and relationship to adjacent structures [10,11]. Multiplanar reconstruction is particularly valuable near the thoracolumbar junction, where a superior lumbar hernia may extend cranially beneath the rib cage, mimicking a diaphragmatic defect. The key distinction is the structure traversed by the herniated contents: a superior lumbar hernia passes through the posterolateral abdominal wall inferior to the 12th rib, whereas a diaphragmatic hernia traverses the diaphragm (Table 1). The tissue plane of the defect and continuity of the diaphragm may therefore be more informative than the final position of the herniated viscera.

**Table 1:** Features distinguishing superior lumbar and diaphragmatic hernias

Feature	Superior Lumbar Hernia	Diaphragmatic Hernia
Location	Superior posterolateral abdominal wall [1-4,6]	Defect through the diaphragm; location varies by hernia type
Anatomic landmarks	12th rib superiorly; internal oblique laterally; quadratus lumborum/paraspinal musculature medially [1,4,6]	Diaphragmatic defect separates abdominal and thoracic compartments
Diaphragm	Intact	Disrupted
Typical contents	Retroperitoneal fat, colon, small bowel, or other abdominal contents [1-4,6]	Stomach, small or large bowel, liver, spleen, or other abdominal viscera, depending on defect location
CT findings	Defect in posterolateral abdominal wall below the 12th rib; contents may extend posteriorly, laterally, or superiorly [6,10,11]	Abdominal viscera traverse a diaphragmatic defect and extend into the thoracic cavity [11,12]

Diagnostic confusion can occur in either direction. Kumar et al. described an acquired diaphragmatic hernia initially interpreted as a lumbar hernia [12], whereas in our patient, imaging suggested a diaphragmatic defect and operative exploration demonstrated a superior posterolateral abdominal wall defect instead. Although the individual muscular boundaries of the Grynfeltt-Lesshaft triangle were not fully documented intraoperatively, the location of the defect and integrity of the diaphragm were consistent with a superior lumbar rather than a diaphragmatic hernia. When this distinction cannot be established preoperatively, operative planning should accommodate both possibilities. In our patient, preparation of both the abdomen and right chest allowed for thoracic intervention if required.

Symptomatic lumbar hernias are generally managed surgically, with mesh reinforcement favored for definitive repair when feasible [2-4]. Open, laparoscopic, robotic, and hybrid approaches have been described, with the choice of technique determined by defect anatomy, tissue quality, ability to achieve adequate mesh overlap and fixation, and clinical circumstances [2-4,13,14]. Minimally invasive approaches may facilitate visualization and mesh placement in selected elective cases; however, acute incarceration or suspected strangulation shifts the immediate priority to reduction and assessment of bowel viability [13,14]. In our patient, markedly dilated bowel limited operative exposure, while the superior posterolateral location of the defect and the patient's body habitus further complicated mesh placement and secure fixation. Primary repair was therefore performed.

Recurrence after lumbar hernia repair is difficult to quantify because of small study populations, heterogeneous techniques, and variable follow-up [2,3]. In our patient, recurrent herniation was documented approximately four months after primary repair. The absence of mesh reinforcement may have contributed to recurrence, although the approximately 5-cm defect and continued mechanical stress from obesity and repetitive coughing may also have played a role. This recurrence illustrates the distinction between emergency treatment of an incarcerated hernia and durable abdominal-wall reconstruction. When definitive mesh reinforcement is not feasible during the index operation, staged reconstruction after recovery may be considered.

This case highlights three practical considerations. First, superior lumbar hernia should remain in the differential diagnosis of a posterolateral thoracoabdominal hernia near the twelfth rib. Second, multiplanar CT evaluation should focus on the tissue plane of the defect and continuity of the diaphragm rather than solely on the final position of the herniated viscera. Finally, emergency reduction and primary closure should be distinguished from definitive abdominal wall reconstruction when operative conditions limit mesh-based repair.

## Conclusions

Superior lumbar hernia is an uncommon posterolateral abdominal wall defect that may rarely present with acute small bowel obstruction due to incarceration. Its proximity to the twelfth rib and the diaphragm can create a diagnostic dilemma with diaphragmatic hernia. This case demonstrates the importance of carefully reviewing multiplanar CT imaging, together with the clinical presentation, to accurately localize the defect and guide operative planning.

When the diagnosis remains uncertain, operative preparation should permit flexibility in surgical approach. Although primary closure may be necessary in selected emergency settings, recurrence in this case underscores the potential benefit of durable mesh-based reconstruction when feasible. Further studies are needed to define optimal repair techniques and strategies to minimize recurrence.
